# Seroepidemiological and parasitological evaluation of the heterogeneity of malaria infection in the Gambia

**DOI:** 10.1186/1475-2875-12-222

**Published:** 2013-07-01

**Authors:** Abraham R Oduro, David J Conway, David Schellenberg, Judith Satoguina, Brian M Greenwood, Kalifa A Bojang

**Affiliations:** 1 Medical Research Council Unit, Atlantic Road, Fajara, Gambia; 2 London School of Hygiene and Tropical Medicine, Keppel St, London WC1E 7HT, UK; 3 Navrongo Health Research Centre, Post Office Box 114, Navrongo, Ghana

**Keywords:** Evaluation, Microheterogeneity, *Plasmodium falciparum*, Parasite prevalence, Serology, The Gambia

## Abstract

**Background:**

As countries make progress in malaria control, transmission may be reduced to such an extent that few cases occur, and identification of the remaining foci of transmission may require a combination of surveillance tools. The study explored the usefulness of parasite prevalence, seroprevalence and model-estimated seroconversion rates for detecting local differences in malaria transmission in a West African country.

**Methods:**

Age-stratified cross-sectional surveys were conducted during the wet season in 2008 and the following dry season in 2009 in The Gambia. In each season, 20 village communities were sampled from six diverse areas throughout the country. A total of 7,586 participants were surveyed, 51% (3,870) during the wet season. Parasites were detected by thick film slide microscopy, and anti-MSP1-_19_ antibodies were detected by ELISA using eluted dried blood from filter papers.

**Results:**

Overall parasite prevalence was 12.4% in the wet season and 2.2% in the dry season, with village-specific parasite prevalence ranging from 1.4 to 45.9% in the wet season and from 0.0 to 13.2% in the dry season. Prevalence was highest in the eastern part of the country. Serological indices also varied between villages, indicating local heterogeneity in transmission, and there was a high correlation between wet and dry season estimates across the villages. The overall prevalence of anti-MSP1_19_ antibodies was similar in the wet (19.5%) and in the dry (19.6%) seasons.

**Conclusion:**

The study illustrates the utility of measuring both parasite prevalence and serological indices for monitoring local variation in malaria transmission, which are more informative than single measures as control intensifies and malaria declines. Measurements of seropositivity have the logistical advantage of being relative stable seasonally so that sampling at any time of year may be conducted.

## Background

Malaria remains the most important tropical infection and continues to pose a major global health challenge [1]. A recent increase in investment for malaria control is leading to changes in the epidemiology of the infection that need to be monitored in order to track progress [2,3]. Effective monitoring will enable the long-term overall impact of control measures on transmission, disease burden and mortality to be determined. It will also help identify localized foci, frequently termed ‘hotspots’, that need targeted intervention in order to drive down transmission even further [4-6]. In areas of low malaria transmission identification, targeting of hotspots of transmission for focussed control efforts will be key to success in malaria elimination [5]. New approaches are required to evaluate the impact of control on transmission at the micro-epidemiological level [7,8], and developing these approaches is now a public health priority.

The entomological inoculation rate (EIR) is a useful measure for assessing impact of interventions on human-mosquito contact, but is highly labour-intensive and lacks precision because of the heterogeneous nature of mosquito distribution [9-11]. The *Plasmodium falciparum* parasite rate (*PfPR*) in children is a more accessible measure but it varies with the level of acquired immunity, access to anti-malarials and season [12-15]. Patent parasitaemia is usually short-lived, and is therefore useful for measuring recent changes in malaria transmission [13-15]. Anti-malarial antibodies, which show less short-term variation in detectable positivity, can track changes in transmission over a longer time. It has recently been shown that estimated rates of seroconversion of anti-malarial antibodies correlate well with EIR as a measure of force of infection, thus making them a robust surrogate measure of transmission intensity [16-19].

The merozoite surface protein 1 (MSP1) is an abundant and important protein. The C-terminal 19 kDa region (MSP1-19) is viewed as a promising vaccine candidate and target of immunity.

Simultaneous analysis of serological and parasitological indices has the potential to discriminate between causes of local geographical variation in endemicity, due to recent implementation of control measures or variation due to longer-term ecological changes. The study explored the usefulness of anti-MSP1-_19_ sero-prevalence, model-estimated seroconversion rates and parasite prevalence for detecting variation in local endemicity in The Gambia, where malaria has been apparently declining.

## Methods

### Study area and setting

The study was conducted in The Gambia which is situated on the coast of West Africa. The Gambia has a subtropical climate with distinct long, dry and short rainy seasons. Annual rainfall averages 920 mm/year in the interior and 1,450 mm/year along the coast. Average annual temperatures ranges from 23 to 27°C along the coast and from 24 to 32°C inland. The landscape is dominated by the basin of the River Gambia and its flood plains. Malaria transmission is seasonal, previously described as hyperendemic, and confined largely to the single, short rainy season; it is strongly associated with the prevailing land cover. The important malaria vectors are members of the *Anopheles gambiae* complex (*An*. *gambiae sensu stricto*, *Anopheles arabiensis*, and *Anopheles melas*); the sporozoite rate and the human blood index have previously been described to be highest in the flood plain of the River Gambia in the middle of the country, and in the eastern part of the country further inland [20-23].

### Study design

**Figure 1:**
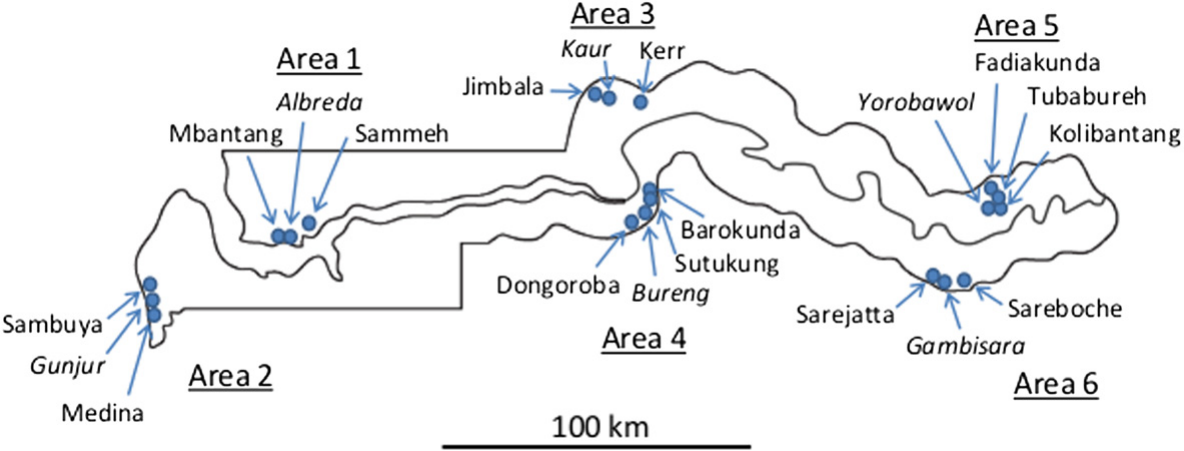
**Map of The Gambia showing the six areas and 20 study villages samples in the wet season (September to November) of 2008 and dry season (March to May) of 2009.** The villages which have health centres are shown in italics.

Details of the study areas (Figure 1) have been presented previously, in a report that included data analysed from health centres [7]. The present study focuses on community-based samples in more detail. In brief, the study involved a series of age-stratified, cross-sectional surveys conducted during the wet season (September to November) of 2008 during which malaria transmission occurred, and during the dry season (March to May) of 2009 during which minimal transmission occurred. Six ecologically diverse areas were selected across the five administrative divisions of The Gambia, including two areas each from the coast, middle and east of the country. One of each pair of study areas was situated to the north and one to the south of the River Gambia. The population sample included people of all age groups and of either sex who were long-term residents and consented to participate. The age distributions of the individuals in the participating 20 villages were comparable.

Participants were enrolled consecutively into pre-defined stratified age groups after witnessed informed consent had been obtained. The same 20 villages and study sampling procedures were used for both the wet and dry season surveys. Different samples of individuals were selected in the wet and dry season surveys. Data collection included administration of a study questionnaire to collect demographic, clinical and socio-economic information. In addition, body weight and axillary temperature were measured.

### Laboratory methods

Five drops of blood were collected from all participants for parasite detection by microscopy, and haemoglobin concentration determination using a Hemocue photometer (Hemocue® Hb 301 Photometer -Leo Diagnostics, Sweden) and three drops of blood were collected onto Whatman 3MM filter paper, which was sealed and stored dry with dessicant at ambient temperature. Reconstituted sera were obtained from the filter paper specimens and tested for anti-MSP-1_19_ IgG antibodies by indirect ELISA using the recombinant blood-stage *P*. *falciparum* antigen MSP-1_19_, employing previously described protocols [17,18]. Duplicate optical densities (ODs) of the ELISA results were averaged and normalized against a positive control. The cut-off for seropositivity was an OD three standard deviations or more above the mean OD obtained in samples from 20 Europeans who had not been exposed to malaria. Malaria antibody reactivity was categorized as seropositive or negative. Estimates of transmission intensity were derived from fitting reverse catalytic models to the age seroprevalence data [16,19]. The Model is: Pt = λ / (λ + ρ) [1-exp (−(λ + ρ)^**t**^)] where P_**t**_ = proportion of seropositives at time (t), λ is seroconversion rate and ρ is the seroreversion rate. The parameter, λ (seroconversion rate), is related to the force of infection [16].

### Data management and analysis

Data were captured using forms designed specifically for this study. All completed forms were checked for internal consistency and queries were resolved before data were double entered using OpenClinica database. All statistical analyses were computed using Stata 11 (9 StataCorp College Station, Texas 77845 USA). All point estimates have interval estimates including the 95% confidence interval, range or interquartile range. Statistical testing involved t-tests, chi-square tests or two sample tests of proportions, and Pearson’s correlation co-efficient analyses. The 95% confidence intervals of proportions were derived from point estimates and sample sizes. All statistical estimations and hypotheses testing were based on parametric methods, and were two sided.

### Ethical approvals

The Gambia Government/Medical Research Council Unit Joint Ethics Committee gave ethical approval for the study after approvals had been obtained from community elders. Witnessed informed consent and, when applicable, child assent were obtained from all study participants.

## Results

### Characteristics of study population

A total of 7,586 participants from 20 villages across the country were studied. Fifty-one percent (3,870) were recruited in the wet season, and 51% (3,834) came from villages to the south of the River Gambia. Overall, 34.2, 32.7 and 33.1% of the participants were recruited from the coastal, middle and eastern areas of the country, respectively. Females and children under five years old constituted 53.1 and 34.6% of study participants, respectively. Average age in months and weight in kg were similar in the wet and dry seasons (respectively 196.8 *vs* 193.8 months, P = 0.54; 31.2 *vs* 31.5 kg, P = 0.49). Mandinkas were the largest participating group in both the wet (58.1%) and dry (55.4%) seasons. Further details on the study population are shown in Table 1.

**Table 1:** **Anti-MSP-1_19_ seropositivity and *Plasmodium falciparum *parasite prevalence tabulated by attributes of the study population**

** Attributes**	**Wet season, % (N)**		**Dry season, % (N)**	
	**Seropositive**	**Parasite positive**	**Seropositive**	**Parasite positive**
Total	19.5 (3823)	12.4 (3870)	19.6 (3716)	2.2 (3707)
**Age groups (years)**				
1-5	5.6 (1095)	10.0 (1211)	7.2 (1193)	1.6 (1095)
6-12	16.0 (751)	16.6 (759)	13.8 (754)	4.5 (753)
13-25	29.2 (764)	18.2 (769)	26.6 (756)	3.3 (755)
>25	38.0 (809)	9.0 (820)	44.2 (783)	0.3 (782)
**Gender**				
Males	18.9 (1629)	12.7 (1797)	18.9 (1610)	2.3 (1754)
Females	22.7 (1888)	12.1 (2063)	23.0 (1778)	2.1 (1953)
**Slept under ITN**				
Yes	19.2 (3311)	11.6 (3348)	18.2 (2934)	1.6 (2931)
No	22.4 (500)	17.5 (508)	24.5 (676)	4.4 (766)
**Ethnicity**				
Fula	26.1 (982)	17.1 (996)	27.8 (1023)	4.3 (1023)
Wolof	18.4 (293)	18.6 (295)	18.1 (276)	1.5 (275)
Mandinka	17.7 (2215)	9.1 (2243)	15.8 (2057)	1.3 (2054)
Others	14.2 (325)	15.3 (236)	18.3 (355)	1.1 (355)
**Settings**				
Coastal	12.4 (1343)	8.8 (1355)	11.3 (1237)	1.2 (1234)
Mid country	23.5 (1225)	9.8 (1236)	21.2 (1245)	1.5 (1244)
East country	23.4 (1255)	18.6 (1279)	26.0 (1229)	3.8 (1229)

Overall parasite prevalence was 12.4% in the wet season and 2.2% in the dry season (OR = 6.4: 95% CI 4.9,8.1; P < 0.0001). Gametocyte carriage rate was 1.5 and 0.2% in the wet and dry seasons, respectively, with the highest carriage rate of 2.2% being recorded in six to 12 year old children in the wet season. Parasite prevalence was similar in males (12.7%) and females (12.1%) in the wet season (OR = 1.1: 95% CI 0.8, 1.2; P = 0.59) and in the dry season (2.3% *vs* 2.1%; OR = 1.1; 95% CI 0.7, 1.7; P = 0.70). Parasite prevalence was lower in children under five years of age than in the older age groups in the wet season (8.5% *vs* 14.4%: OR = 0.6: 95% CI 0.4,0.7; P <0.0001) and in the dry season (1.3% *vs* 2.6%; OR= 0.5: 95% CI 0.2, 0.8; P = 0.007).

The prevalence of fever (axillary temperature ≥37.5°C) was 5.5% in the wet season and 2.2% in the dry season (OR = 2.6; 95% CI 2.0, 3.3; P < 0.0001). Approximately 25% (53/161) of fevers were associated with malaria infection (positive microscopy) in the wet season, compared with only 4% (3/80) of fevers were associated with malaria infection in the dry season (OR = 8.8: 95% CI 2.5, 36.0; P < 0.0001).

Mean haemoglobin (Hb) concentration was significantly higher (P < 0.0001) in the dry season (11.61 g/dl; SD = 1.85 ) than in the wet season (11.05 g/dl; SD = 2.06). The prevalence of anaemia (defined as Hb concentration ≤8 g/dl) was 7.4 and 3.4% in the wet and dry seasons, respectively. A low Hb concentration was associated with being female, parasitaemic, age under six years and living in the eastern part of The Gambia. Reported insecticide-treated bed net (ITN) use during community surveys was 86% in the wet season and 73% in the dry season, respectively, and was highest in children under two years and lowest in adolescents and young adults (13–25 year-old group). Overall, males were less likely than females to sleep under a mosquito net (OR = 1.6; 95% CI 1.3, 1.9; P < 0.001) in the wet season. Adolescent and young adult males were much less likely to sleep under a mosquito net than females of the same age (OR = 3.1, 95% CI 2.1, 4.4; P < 0.001). Participants reporting to be using treated nets had a significantly lower risk of malaria infection than those reporting to be non-users (OR = 0.6; 95% CI 0.5,0.9; P = 0.007).

The prevalence of anti-MSP1_19_ antibodies was similar in the wet (19.5%) and in the dry (19.6%) season (P = 0.91). Seroprevalence increased with age (χ^2^ for trend; P < 0.0001) and was lower in males 17.5% (618/3,531) than in females 21.3% (852/3,995) (OR = 0.78: 95% CI; 0.70,0.88; P < 0.0001). Multivariate logistic regression analyses adjusting for age, study area and season of survey showed that female gender (OR = 1.17: 95% CI 1.03, 1.32; P < 0.05), Fula ethnicity (OR = 1.07: 95% CI 1.03, 1.11; P < 0.05) and active malaria infection (OR = 2.30: 95% CI 1.87,2.81; P < 0.001) were independently associated with seropositivity. Table 1 summarizes parasite prevalence and seroprevalence in all study participants.

### Local variations in seroprevalence and parasite prevalence

**Figure 2:**
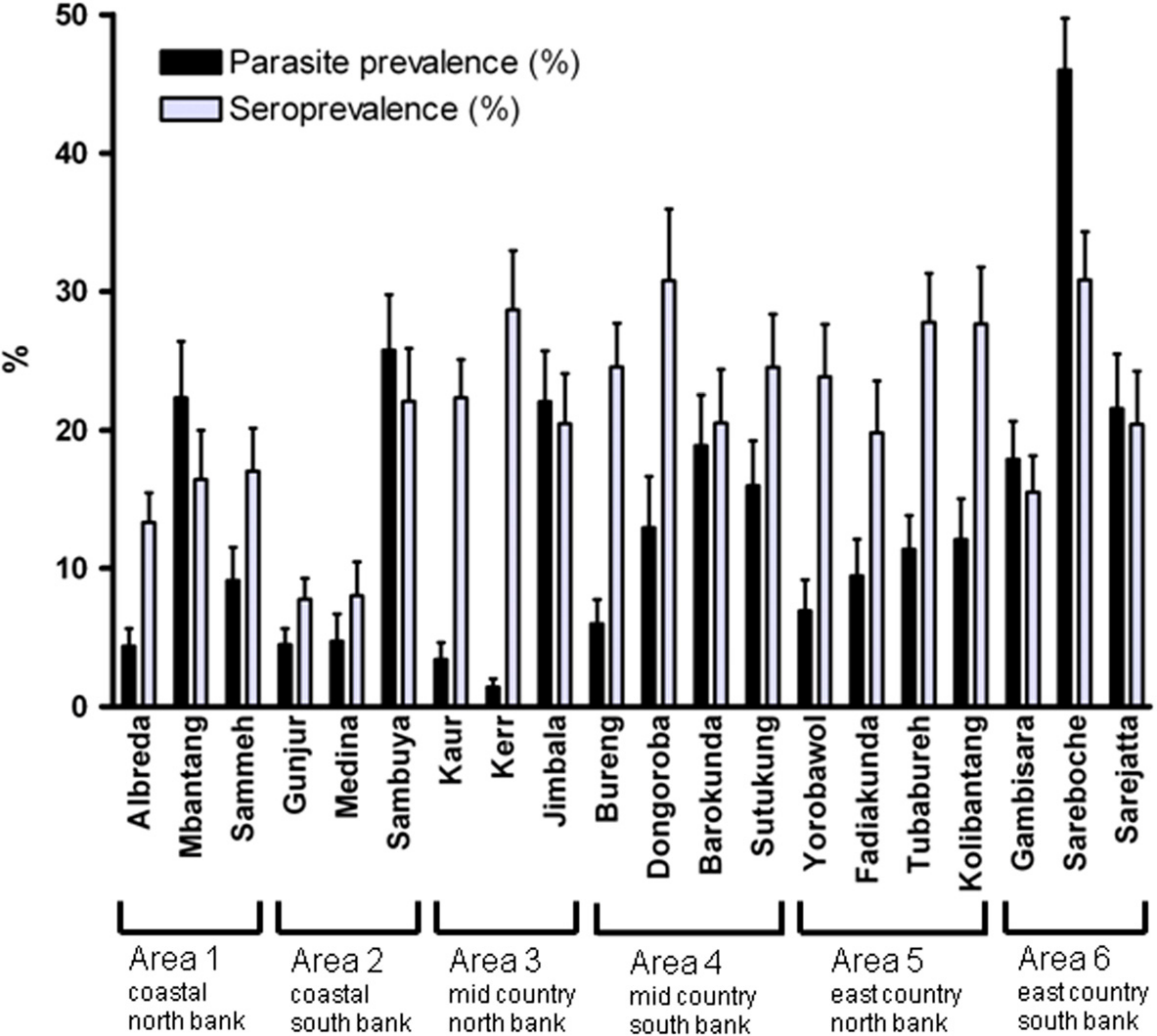
**Parasite prevalence (%, 95% CI) and anti-MSP1_19_ seroprevalence (%, 95% CI) in each of the 20 study villages from six different areas of The Gambia, from the wet season sampling in 2008.** Sample sizes and numbers, as well as tests for inter-village heterogeneity within areas, are given in Additional files 1 and 2.

Significant inter- and intra-area heterogeneity in malaria infection was detected (Figure 2 and Additional file 1). The overall parasite prevalence in the wet season was 15.9% (310/1,954) in villages on the south of the River Gambia and 8.8% (168/1,916) in villages north of the river (OR = 2.0; 95% CI 1.6, 2.4; P < 0.0001). Similar findings (parasite prevalence to the south 2.9% *vs* 1.4% to the north of the river) were observed in the dry season (OR = 2.2 95% CI 1.3, 3.6; P = 0.001). The area east of the country and south of the river had the highest parasite prevalence (28.9%) whereas the central part of the country to the north of the river had the lowest prevalence (7.7%).

Village-specific parasite prevalence ranged from 1.4 to 45.9% in the wet season and from 0.0 to 13.2% in the dry season. Correlation between parasite prevalence in the wet and in the dry season by village was strong (R^2^ = 0.741). Half (10/20) of the villages studied had a parasite prevalence of <10% in the wet season, and the prevalence was significantly heterogeneous among villages within all six areas (Additional file 1), except in the east of the country to the north of the river where the variation was not statistically significant. In all of the six different areas, the lowest prevalence rates were in villages that had health centres (Figures 1 and 2).

**Figure 3:**
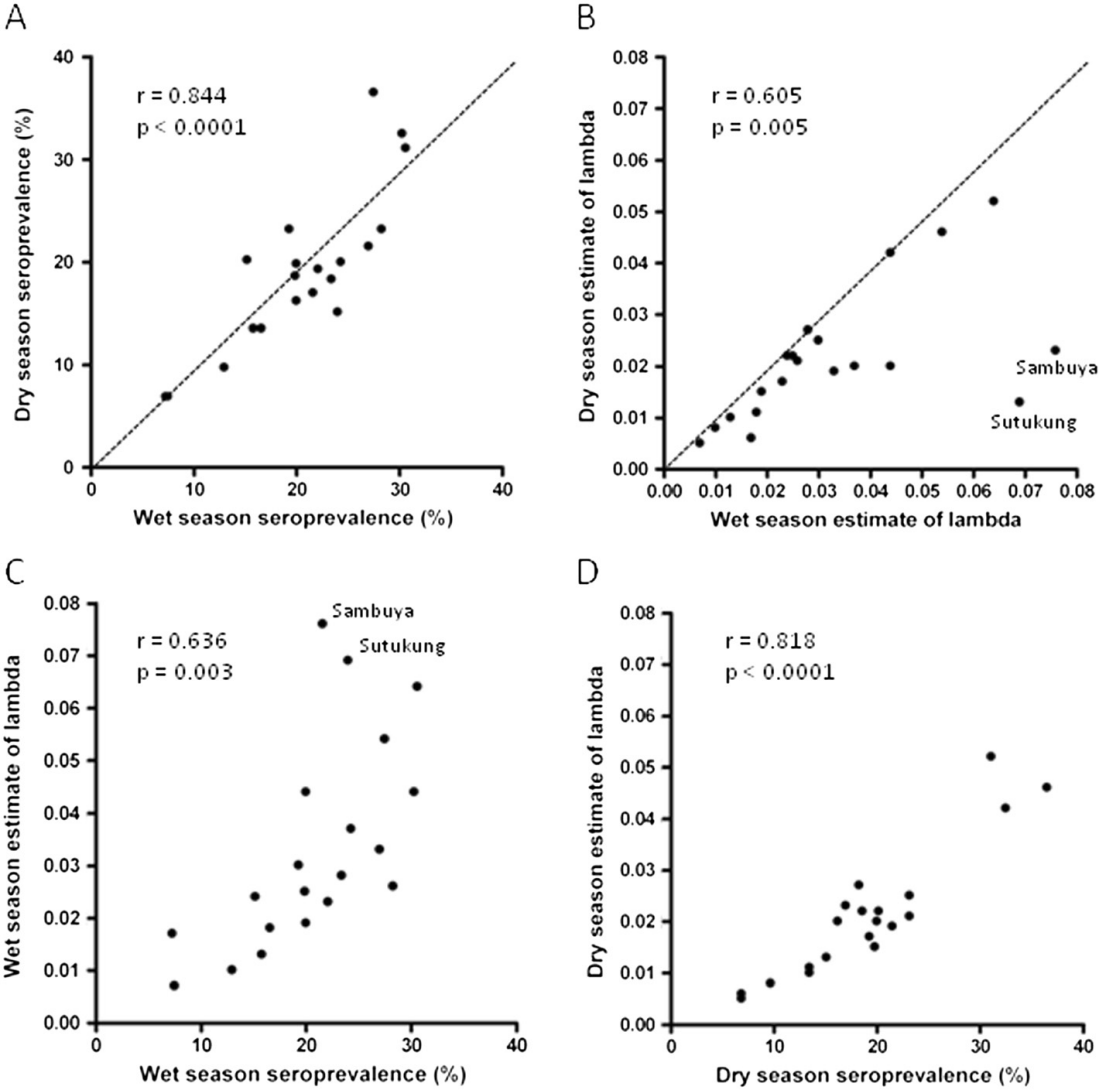
**Correlations among serological measures across the 20 different villages sampled throughout The Gambia.** (**A**) Seroprevalence measurements from the wet and dry seasons are similar (dotted line shows x = y) and very highly correlated. (**B**) Derived lambda parameters from the age-seroprevalence distributions tend to be slightly higher for the wet season estimate, with outlying data for two labelled villages. (**C**) Wet season correlation between seroprevalence and derived lambda parameter, showing two labelled villages with an elevated lambda estimate. (**D**) Dry season correlation between seroprevalence and derived lambda parameter. Estimates of the lambda parameter with 95% confidence intervals for each season are given in full in Additional file 3.

Variations in seroprevalence among villages are presented in Figures 2 and 3 (with further details in Additional file 2). Seroprevalence was very similar in both seasons (Figure 3A). There were variations between villages within each catchment area, although this was not statistically significant for all comparisons (Additional file 2). Seroprevalence varied from approximately 15% in the coastal areas to 24% in the east of the country in the wet season (OR = 0.52: 95% CI 0.39, 0.70; P < 0.0001). Similar findings were observed in the dry season, from 10% in the coastal areas to 27% in the east (OR = 0.29: 95% CI 0.21, 0.41; P < 0.0001). The lower endemicity in areas near the coast is consistent with variation previously described in The Gambia [24].

The within-area test of heterogeneity for seroprevalence was significant in four areas in the dry season but in only two areas in the wet season, probably due to the influence of new malaria infections during the wet season. Seroprevalence was higher in participants with active malaria infection than in those without (32.9% *vs* 17.7%; P < 0.001). Seroprevalence was lowest in children and increased with age in all areas and in each season (χ^2^ for trend = P < 0.001) indicative of cumulative acquisition of antibodies with infection experience, as expected.

The seroconversion rate estimates for each of the 20 villages are shown in Additional file 3. These estimates were similar in the wet and dry season sampling for most villages but were elevated in the wet season for two of the villages (Sutukung and Sambuya, Figure 3B). Among all villages, there were strong correlations between seroprevalence and estimates of lambda in both wet season (Figure 3C) and dry season (Figure 3D), except for the apparently elevated values of lambda in two of the villages in the wet season (Figure 3C). Seroconversion rate (lambda) estimates from the age-seroprevalence curves for the six different study areas in the wet season are shown in Additional file 4, showing no major step change in any of the curves.

### Local variation in parasite prevalence, seroprevalence and access to malaria control

The study consider variation between villages in each of the six areas in relation to features of the villages (Figure 2). Seroprevalence in the three study villages in Area 1 was similar, suggesting similar past malaria exposure. However, parasite prevalence was higher in the village of Mbantang than in the two other villages, possibly because ITN usage was lower in Mbantang compared to Sammeh and Albreda (OR = 0.13: 95% CI 0.06, 0.30; P < 0.0001) and because residents of Albreda, where the health centre is located, may have better access to anti-malarial treatment. In Area 2, both seroprevalence and parasite prevalence were higher in Sambuya than in Gunjur or Medina village. Sambuya is a new settlement surrounded by bush vegetation, suggesting it might benefit from a targeted malaria intervention like indoor residual spraying, while Gunjur has the benefit of being the location of the health centre. In Area 3, seroprevalence in the the three study villages was similar, but parasite prevalence in Jimbala was higher than in Kaur or Kerr, probably due to lower access or uptake of interventions. This view is supported by the observation that reported use of ITNs was significantly lower in Jimbala (59.3%) compared to Kerr Auldeh (83.2%), and Kaur (88.2%) where the area health centre is located (P < 0.0001). In Area 4, seroprevalence in the four study villages was similar. Parasite prevalence was generally low, perhaps due to a high overall use of ITNs, and lowest in Bureng, where the area health centre is located. In Area 5, seroprevalence in the four study villages was similar, and parasite prevalence did not differ significantly between villages although it was lowest in Yorobowal where the health centre is located. In Area 6, Sareboche village had a higher seroprevalence (30.6%) than Gambisara (15.2%) or Sarejatta (19.9%) (P < 0.0001), and parasite prevalence was also higher in Sareboche (45.9%) than in Gambisara (17.6%) or Sarejatta (21.0%) (P <0.0001), indicating persistent high malaria transmission in Sareboche village which is located furthest from the health centre in Gambisara.

## Discussion

Malaria control strategies at country level are often recommended as a universal solution to fit all endemic settings even though intensity of transmission may vary locally [25]. New approaches are required for rapid assessment of the micro-epidemiological situations to allow for more targeted interventions [7,8] because control of infection in major foci remains key to long-term successes of malaria control and elimination [4-6]. In this study, the utility of using parasite and serological measures to detect variation in local transmission of malaria in neighbouring communities is examined. It illustrates that concurrent use of both measures can help in determining whether local differences are due to differences in exposure over a prolonged period or to more recent changes in access to treatment or implementation of preventive measures such as ITNs.

In four out of six different areas studied in The Gambia in the wet season, seroprevalence did not differ significantly among local study villages, whereas in these same areas parasite prevalence did vary significantly among the villages (Figure 2). This suggests that differences in access or uptake of available interventions were dominant factors influencing variation in parasite prevalence. This is supported by the observation that reported ITN use was always lower in villages with higher prevalence compared with those with lower prevalence, and by the fact that villages with health centres had lowest parasite prevalence. In the two remaining areas, seroprevalence in the catchment villages was not homogenous, and parasite prevalence correlated well with seroprevalence suggesting that differences in parasite prevalence were more due to variation in local transmission intensity than to differences in access to antimalarials. This finding is consistent with previous studies that have shown differences in transmission risk between adjoining villages [5,25].

Village-specific seroprevalence and modelled seroconversion rates were highly correlated, and similar in both wet and dry seasons, suggesting that for application of either serological measurement of exposure it would be suitable to sample in either the wet or dry season. It also indicates that with an age-structured sampling such as that performed here, either seroprevalence or derived lambda estimate of seroconversion appears to be highly informative. This confirms the utility of serological analysis as an adjunct measure for describing changes in the micro-epidemiology of malaria [16].

## Conclusion

The utility of serology and parasitology for monitoring local heterogeneity of malaria transmission and impact of interventions is supported by this study. The data add to understanding of variation in malaria endemicity in The Gambia, a country in which many studies have been conducted previously and in which malaria incidence has declined [26]. The combination of parasite prevalence and serological analyses in community surveys may be increasingly informative if control intensifies and malaria declines further.

## Competing interests

The authors declare that they have no competing interests.

## Authors’ contributions

ARO, DC and KB: conception and design, data acquisition, analysis and interpretation and manuscript preparation. JS: data acquisition, analysis and interpretation, and manuscript preparation. DS and BG: conception and design, interpretation of data, and manuscript preparation. All authors read and approved the final manuscript.

## Supplementary Material

Additional file 1 **Area, village and seasonal variation in malaria parasite prevalence in The Gambia.** The table summarizes the seasonal variation in malaria parasite prevalence in The Gambia by village and and study area. Click here for file

Additional file 2 **Area, village and seasonal variation in anti-MSP-1_19_ seroprevalence in The Gambia.** The data provided describes the seasonal variation in in anti-MSP-1_19_ seroprevalence in The Gambia. Click here for file

Additional file 3 **Variation in modelled seroconversion rate estimates (λ) by study area, village and season.** The table summarizes the seasonal variation in in the modelled seroconversion rate estimates (λ) in The Gambia by village and and study area. Click here for file

Additional file 4 **Age seroprevalence of anti-MSP1_19_ antibodies in the community surveys in the six different areas sampled in The Gambia in the wet season of 2008.** Modelled estimates of seroconversion rates and 95% confidence intervals. Click here for file
