# Supplementary material for: Seroepidemiological and parasitological evaluation of the heterogeneity of malaria infection in the Gambia
Source: Malar J. 2013 Jul 1;12:222. doi: 10.1186/1475-2875-12-222 (PMC3701490; doi:10.1186/1475-2875-12-222)
Supplement: Additional file 1 — Area, village and seasonal variation in malaria parasite prevalence in The Gambia. The table summarizes the seasonal variation in malaria parasite prevalence in The Gambia by village and and study area. [file 1475-2875-12-222-S1.docx]

**Additional file 1 Area, village and seasonal variation in malaria parasite prevalence in The Gambia**

| **Settings** Bank of River Gambia | Study villages | **Parasite prevalence, % (N)** | | |
| --- | --- | --- | --- | --- |
|  |  | West season | Dry season | |
| **Coastal (North bank)** | Albreda | 4.0 (324) | | 0.8 (250) |
|  | Mbantang | 21.8 (133) | | 0.0 (121) |
|  | Sammeh | 8.6 (187) | | 1.7 (239) |
|  | χ^2^ (P-value) | 36.6 (P<0.001) | | 2.5 (P=0.292) |
|  |  |  | |  |
| **Coastal (South bank)** | Gunjur | 4.2 (409) | | 0.3 (405) |
|  | Medina | 4.0 (152) | | 3.8 (106) |
|  | Sambuya | 25.3 (150) | | 3.5 (113) |
|  | χ^2^ (P-value) | 68.6 (P<0.001) | | 11.6 (P=0.002) |
|  |  |  | |  |
| **Mid country (North bank)** | Kaur | 3.0 (297) | | 0.7 (303) |
|  | Kerr | 1.4 (139) | | 1.3 (151) |
|  | Jimbala | 21.6 (162) | | 2.6 (156) |
|  | χ^2^ (P-value) | 60.9 (P<0.001) | | 2.9(P=0.236) |
|  |  |  | |  |
| **Mid country (South bank)** | Bureng | 5.5 (235) | | 0.5 (204) |
|  | Dongoroba | 12.0 (100) | | 1.6 (128) |
|  | Barokunda | 18.3 (142) | | 3.3 (151) |
|  | Sutukung | 15.4 (156) | | 1.3 (153) |
|  | χ^2^ (P-value) | 16.5 (P<0.001) | | 4.6 (P=0.206) |
|  |  |  | |  |
| **East country (North bank)** | Yorobawol | 6.3 (160) | | 0.0 (121) |
|  | Fadiakunda | 8.7 (149) | | 0.7 (140) |
|  | Tuba-bureh | 10.9 (211) | | 3.7 (217) |
|  | Kolibantang | 11.4 (149) | | 1.5 (130) |
|  | χ^2^ (P-value) | 3.3 (P=0.36) | | 7.5(P=0.056) |
|  |  |  | |  |
| **East country (South bank)** | Gambisara | 17.6 (245) | | 0.4 (236) |
|  | Sareboche | 46.0(222) | | 13.2 (243) |
|  | Sarejatta | 21.0 (138) | | 2.4( 140) |
|  | χ^2^ (P-value) | 51.1 (P<0.001) | | 40.0 (P<0.0001) |
|  |  |  | |  |
