# Supplementary material for: Seroepidemiological and parasitological evaluation of the heterogeneity of malaria infection in the Gambia
Source: Malar J. 2013 Jul 1;12:222. doi: 10.1186/1475-2875-12-222 (PMC3701490; doi:10.1186/1475-2875-12-222)
Supplement: Additional file 2 — Area, village and seasonal variation in anti-MSP-119 seroprevalence in The Gambia. The data provided describes the seasonal variation in in anti-MSP-119 seroprevalence in The Gambia. [file 1475-2875-12-222-S2.docx]

**Additional file 2 Area, village and seasonal variation in anti-MSP-1_19_ seroprevalence in The Gambia**

| **Settings** Bank of River Gambia | Study villages | **Anti-MSP-1_19_ seroprevalence, % ( N)** | |
| --- | --- | --- | --- |
|  |  | Wet season | Dry season |
| **Coastal (North bank)** | Albreda | 13.0 (322) | 9.7 (248) |
|  | Mbantang | 15.8 (133) | 13.5 (119) |
|  | Sammeh | 16.6(187) | 13.5 (237) |
|  | χ^2^ (P-value) | 1.4 (P=0.50) | 2.02 (P=0.36) |
|  |  |  |  |
| **Coastal (South bank)** | Gunjur | 7.5 (402) | 6.9 (405) |
|  | Medina | 7.3 (151) | 6.9 (102) |
|  | Sambuya | 21.6 (148) | 17.0 (112) |
|  | χ^2^ (P-value) | 25.3 (P<0.0001) | 11.7 (P=0.002) |
|  |  |  |  |
| **Mid country (North bank)** | Kaur | 22.1 (294) | 19.3 (301) |
|  | Kerr | 28.3 (138) | 23.2 (151) |
|  | Jimbala | 20.0 (160) | 19.8 (157) |
|  | χ^2^ (P-value) | 3.1 (P=0.211) | 1.0 (P=0.607) |
|  |  |  |  |
| **Mid country (South bank)** | Bureng | 24.3 (235) | 20.0 (200) |
|  | Dongoroba | 30.3 (99) | 32.5 (126) |
|  | Barokunda | 20.0 (140) | 16.2 (148) |
|  | Sutukung | 24.0 (154) | 15.1 (152) |
|  | χ^2^ (P-value) | 3.4 (P=0.339) | 15.6(P=0.001) |
|  |  |  |  |
| **East country (North bank)** | Yorobawol | 23.4 (158) | 18.3 (120) |
|  | Fadiakunda | 19.3 (145) | 23.2 (138) |
|  | Tuba-bureh | 27.5 (204) | 36.5 (214) |
|  | Kolibantang | 27.0 (146) | 21.5 (130) |
|  | χ^2^ (P-value) | 3.8(P=0.282) | 17.4(P=0.0005) |
|  |  |  |  |
| **East country (South bank)** | Gambisara | 15.2 (244) | 20.2 (233) |
|  | Sareboche | 30.6 (219) | 31.1 (241) |
|  | Sarejatta | 19.9 (136) | 18.6 (140) |
|  | χ^2^ (P-value) | 16.5(P<0.0002) | 10.8(P=0.004) |
|  |  |  |  |
