# Supplementary material for: Seroepidemiological and parasitological evaluation of the heterogeneity of malaria infection in the Gambia
Source: Malar J. 2013 Jul 1;12:222. doi: 10.1186/1475-2875-12-222 (PMC3701490; doi:10.1186/1475-2875-12-222)
Supplement: Additional file 3 — Variation in modelled seroconversion rate estimates (λ) by study area, village and season. The table summarizes the seasonal variation in in the modelled seroconversion rate estimates (λ) in The Gambia by village and and study area. [file 1475-2875-12-222-S3.docx]

**Additional file 3 Variation in modelled seroconversion rate estimates (λ) by study area, village and season**

| **Settings**  Bank of River Gambia | Study villages | **Seroconversion rates (95% CI)** | |
| --- | --- | --- | --- |
|  |  | Wet season | Dry season |
| **Coastal**  **(North bank)** | Albreda | 0.010 (0.007,0.013) | 0.008 (0.005,0.011) |
|  | Mbantang | 0.013 (0.005,0.031) | 0.010 (0.005,0.015) |
|  | Sammeh | 0.018 (0.009,0.034) | 0.011 (0.006,0.020) |
|  |  |  |  |
| **Coastal**  **(South bank)** | Gunjur | 0.007 (0.003,0.012) | 0.005 (0.002,0.008) |
|  | Medina | 0.017 (0.004,0.064) | 0.006 (0.001,0.025) |
|  | Sambuya | 0.076 (0.031,0.185) | 0.023 (0.006,0.079) |
|  |  |  |  |
| **Mid country**  **(North bank)** | Kaur | 0.023 (0.014,0.037) | 0.017 (0.011,0.026) |
|  | Kerr | 0.026 (0.014,0.043) | 0.02 1(0.011,0.036) |
|  | Jimbala | 0.019 (0.009,0.036) | 0.015 (0.010,0.022) |
|  |  |  |  |
| **Mid country**  **(South bank)** | Bureng | 0.037 (0.022,0.059) | 0.020 (0.011,0.034) |
|  | Dongoroba | 0.044 (0.024,0.080) | 0.042 (0.026,0.068) |
|  | Barokunda | 0.044 (0.018,0.100) | 0.020 (0.009,0.041) |
|  | Sutukung | 0.069 (0.029,0.164) | 0.013 (0.006,0.026) |
|  |  |  |  |
| **East country**  **(Northb ank)** | Yorobawol | 0.028 (0.015,0.048) | 0.027 (0.012, 0.059) |
|  | Fadiakunda | 0.030 (0.017,0.052) | 0.025 (0.013,0.044) |
|  | Tuba-bureh | 0.054 (0.030,0.095) | 0.046 (0.032,0.065) |
|  | Kolibantang | 0.033 (0.017,0.062) | 0.019 (0.010,0.034) |
|  |  |  |  |
| **East country**  **(South bank)** | Gambisara | 0.024 (0.011,0.049) | 0.022 (0.012,0.039) |
|  | Sareboche | 0.064 (0.035,0.114) | 0.052 (0.032,0.084) |
|  | Sarejatta | 0.025 (0.011,0.053) | 0.022 (0.009,0.050) |
|  |  |  |  |
