# Supplementary figures and images for: Seroepidemiological and parasitological evaluation of the heterogeneity of malaria infection in the Gambia
Source: Malar J. 2013 Jul 1;12:222. doi: 10.1186/1475-2875-12-222 (PMC3701490; doi:10.1186/1475-2875-12-222)

Area 1

SCR = 0.012 (0.008, 0.017)

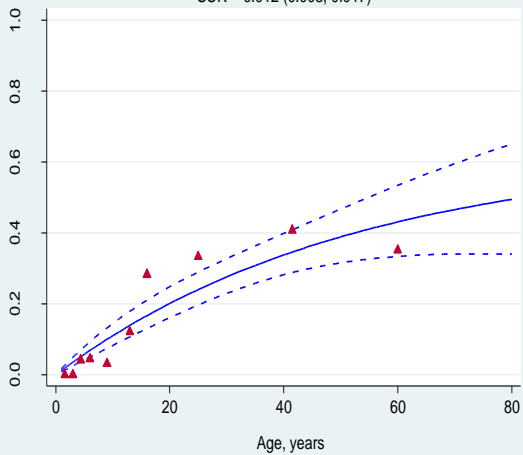

Area 2

SCR=0.019 (0.011,0.032)

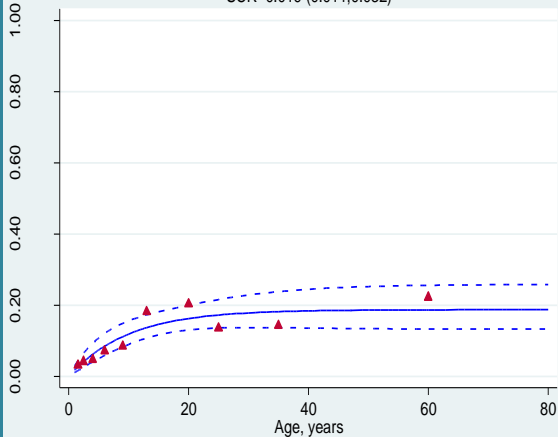

Area 3

SCR=0.022 (0.016,0.029)

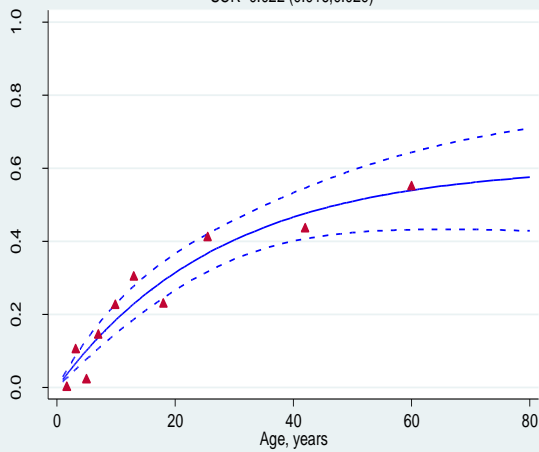

Area 4

SCR=0.042 (0.031,0.058)

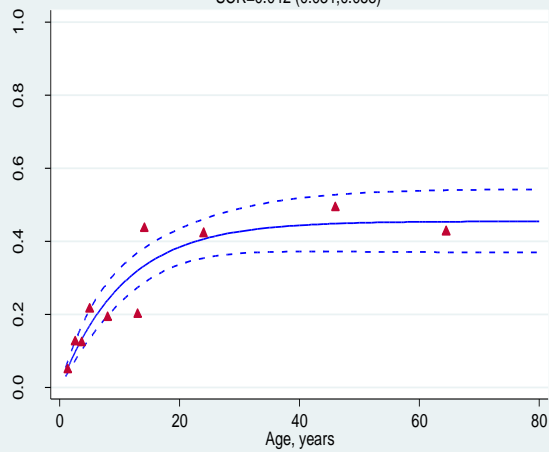

Area 5

SCR=0.036 (0.027,0.047)

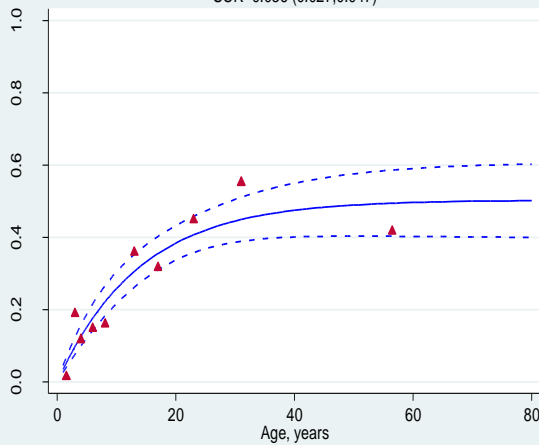

Area 6

SCR=0.039 (0.027,0.057)

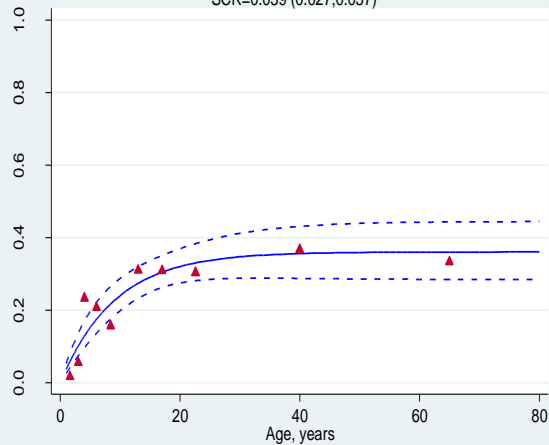

Supplement: Additional file 4 — Age seroprevalence of anti-MSP119 antibodies in the community surveys in the six different areas sampled in The Gambia in the wet season of 2008. Modelled estimates of seroconversion rates and 95% confidence intervals. [file 1475-2875-12-222-S4.pdf]
